# Albumin-mediated “Unlocking” of supramolecular prodrug-like nanozymes toward selective imaging-guided phototherapy†

**DOI:** 10.1039/d2sc02025d

**Published:** 2022-06-06

**Authors:** Jingjing Han, Haidong Li, Luyang Zhao, Gyoungmi Kim, Yahui Chen, Xuehai Yan, Juyoung Yoon

**Affiliations:** Department of Chemistry and Nanoscience, Ewha Womans University Seoul 03760 Republic of Korea jyoon@ewha.ac.kr https://of2m.ewha.ac.kr; School of Bioengineering, Dalian University of Technology Dalian 116024 P. R. China; State Key Laboratory of Biochemical Engineering, Institute of Process Engineering, Chinese Academy of Sciences Beijing 100190 P. R. China yanxh@ipe.ac.cn https://www.yan-assembly.org; New and Renewable Energy Research Center, Ewha Womans University Seoul 03760 Republic of Korea; Center for Mesoscience, Institute of Process Engineering, Chinese Academy of Sciences 100190 Beijing P. R. China

## Abstract

Construction of an activatable photosensitizer and integration into an adaptive nanozyme during phototherapy without producing off-target toxicity remains a challenge. Herein, we have fabricated a prodrug-like supramolecular nanozyme based on a metallic-curcumin and cyanine co-assembly. The albumin-mediated phenol AOH group transformation of nanozyme changes its adjustable oxygen stress from negative superoxide dismutase-like activity of ROS-scavenging to positive photo oxidase activity with an ROS-amplifying capacity. It further increases the depth penetration of a nanozyme in a tumor spheroid, selectively targeting tumorous phototherapy. It also triggers a signal in targeted tumor cells and helps increase cancer cell ablation. This work suggests new options for development of activatable supramolecular nanozymes and provides a synergetic prodrug-like nanozyme strategy for early diagnosis and preclinical phototherapeutics.

## Abstract 2

An adaptive nanozyme without producing off-target toxicity has been successfully applied in phototherapy.

## Graphical abstract

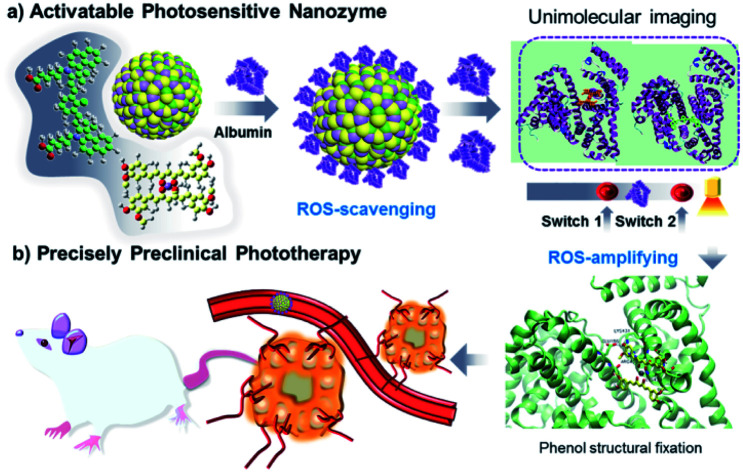

## Introduction

Emerging nanozyme-based phototherapeutics has shown superiority in improvement of therapeutic efficiency over natural enzymes, but it is unknown how to precisely direct its activity in tumors without producing off-target toxicity.^1–6^ Despite achievements in inorganic catalysts (*e.g.*, Fe_3_O_4_), metal complexes,^7,8^ and singlet atom catalysts,^9–11^ differences in activation specificity between nanozymes and natural enzymes suppress their selective therapeutics.^1,12–15^ In living systems, activating a zymogen with signal molecules helps automatically customize the species to affect a targeted area. Cellular systems can activate biorthogonal reactions on-demand for targeting of tumour areas, but it is not simple to visualize.^16,17^ The therapeutic agents are metal-free nanozyme-activated prodrugs to minimize side effects,^18^ although an additional prodrug (*e.g.*, indole-3-acetic acid) is needed in the porous structure.^19,20^ With regard to the available therapeutics, a photo-trigger is superior to other activation switches (*e.g.*, light, heat,^21^ pH, enzymes) due to its unparalleled controllability, noninvasiveness, and high spatial-temporal resolution. Moreover, integration of early diagnostic molecular and therapeutic nanozyme agents produces photoactivatable nanozymes with double “lock” and benefits clinical safety.^22–24^ Therefore, the most anticipated achievement is to fabricate an activatable photosensitive prodrug-like nanozyme to resolve safety with increased selectivity.^25^

A supramolecular assembly was used to construct nanozymes and replicate activatable enzymatic catalysis.^26–29^ Enzymes,^5^ extracellular MMP-2,^30^ and redox-driven disassemblies^31,32^ served as supramolecular activation systems. Among them, non-enzymatic proteins activated the enzyme *via* release of a supramolecular/agarose hydrogel composite-embedded RNase in a hydrogel-transformed solution.^33^ Acid-activatable transmorphic fibres highlighted the dynamic morphology control of molecular assemblies based on deprotonated or protonated peptide-porphyrin.^34^ Before being activated or transformed by acid, these fibres maintained a small amount of reactive oxygen species (ROS). Adding quenchers to adjacent photosensitizers covalently required complicated molecular design, time-consuming synthesis, high cost, and unwanted toxicity.^35–37^ In order to increase the selectivity of the supramolecular nanozyme, scavenging the ROS before activation provided protection, as with activating a zymogen *via* biomarkers and generating singlet oxygen, satisfying targeted phototherapy requirements.

Curcumin, an FDA-approved lipophilic polyphenolic com-pound has antiproliferative, antimetastatic, and antiangiogenic activities in addition to superior biocompatibility.^38–44^ Its hydrogen donation capacity promotes several enzymatic reactions.^45^ However, its rapid degradation, poor solubility, and insufficiency of singlet oxygen impede its photodynamic use as an activatable efficient photosensitive building block.^46–49^ Cyanine has been selected as a multifunctional building block due to its strong absorbance, deep tissue penetration of light, high photodynamic activity, and possible diagnosis capabilities. However, it is insoluble and readily photodegradable.^15,50^ Albumin is not only a biomarker for cancer diagnosis and a carrier,^51–54^ but it may also help curcumin and cyanine stay stable *in vivo*, regulate the generation of reactive oxygen species, and enhance visualization and the efficiency of tumor treatment.^55–61^ It could be used as one “lock” to achieve selective imaging-guided phototherapy. Amphiphilic polypeptide nano-assemblies^62^ for binding induced disassembly and the resulting increased binding sites could also provide options as photosensitizers with the increased depth of penetration and high tumour specificity.

**Scheme 1:**
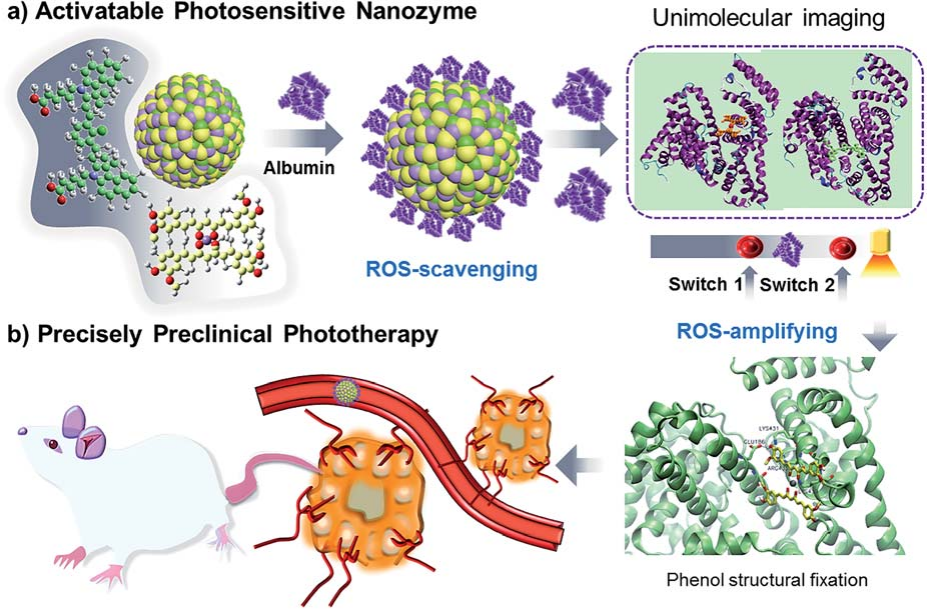
Activatable nanozymes vary from ROS-scavenging to oxidase-like mimics (in targeted tumor spheroids) for precise preclinical photodynamic therapy.

Thus, we have designed a supramolecular nanozyme through an assembly of supramolecular cyanine and Mn–curcumin (Scheme 1). The model mimics the zymogen activation process of the SOD-like enzyme mimic of disproportionation of a superoxide radical anion. This is followed by phenol structural fixation through sufficient albumin, generating singlet oxygen that activated a fluorescent signal through an albumin-mediated unlocking mechanism. Finally, the system avoids off-target toxicity in normal tissues and exhibits high therapy efficiency with regard to tumour cell inhibition. Methods for activating a supramolecular nanozyme minimize the off-target toxicity in cancer treatment for precise photodynamic therapy.

## Results and discussion

Curcumin's photodynamic use is hampered by its rapid degradation, poor solubility, and inadequacy. Curcumin's easy keto–enol tautomerism has made it too sensitive to solvent and pH, as well as resulting in uncontrolled reactive oxygen species.^63^ Low-toxicity Mn(ii) ions not only act as a catalytic mediator but also act as a structural stabilizer by controlling the transformation.^64–66^ As a result, we make an Mn–curcumin (1 : 2) complex through the introduction of Mn(ii) in curcumin (Fig. S1 and S7†). Adding Mn(ii) ions limit keto–enol tautomerism and increase the stability of curcumin by enhancing hydrophobic effect. We also synthesize one amphipathic cyanine compound, IRCOOH, through a two-step reaction (Fig. S2†) to increase the binding capacity with Mn–curcumin. HRMS and ^1^H NMR confirmed the structures of IRCOOH, as shown in the ESI (Fig. S3–S6†).

**Fig. 1:**
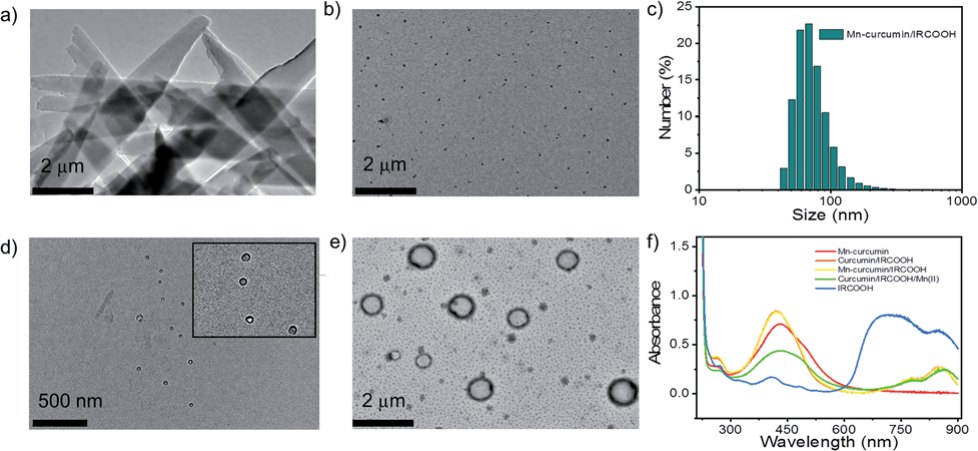
(a) TEM images of Mn–curcumin crystals and (b) Mn–curcumin/IRCOOH NPs and (c) their hydrodynamic size as measured by DLS (images on the right). (d) and (e) TEM images of activatable nanozymes with different albumin concentrations (12 μM and 60 μM, respectively). (f) UV-visible spectrum of Mn–curcumin, IRCOOH NPs, curcumin/IRCOOH NPs, Mn–curcumin/IRCOOH NPs, and curcumin/IRCOOH/Mn(ii) NPs.

In a typical assembly process, IRCOOH (10 mg mL^−1^) and Mn–curcumin (5 mg mL^−1^) were dissolved in a solution of dimethyl sulfoxide and ethanol, respectively. Huge Mn–curcumin crystals were produced in an aqueous solution containing only Mn–curcumin (Fig. 1a). While after mixing 10 μL IRCOOH and 50 μL Mn–curcumin at the final concentration of 100 μg mL^−1^ and 250 μg mL^−1^, respectively, we could get stable but protein-activatable nanoparticles. The TEM images of Mn–curcumin/IRCOOH nanoparticles (NPs) showed that they are about 75 nm, which is consistent with the reported size distribution (75.38 ± 28, PDI: 0.234) measured by DLS (Fig. 1b and c). Mn–curcumin/IRCOOH nanoparticles (NPs) after aging for 24 hours remain stable (Fig. S8†). Although the size distribution increases (84.63 ± 32.65, PDI: 0.208, −41.4 mV), the PDI value proves that the Mn–curcumin/IRCOOH NPs still keep stable for further investigation.

Serum albumin is not only a biomarker for cancer diagnosis,^51^ but it may also help curcumin stay stable *in vivo* by preventing it from degrading quickly.^55–57^ As a result, we chose serum albumin as one phototherapy “lock” for precisely controlling the formation of reactive oxygen species. Nanoparticles' albumin- and photo-responsive characteristics could give photodynamic phototherapy a double-dock in terms of selectivity. A nanoparticle model (Mn–curcumin/IRCOOH) was used to explore the albumin response in different scenarios when using albumin as a bio-signaling molecule. When meeting a slightly increased concentration of albumin (12 μM), the Mn–curcumin/IRCOOH NPs were surrounded by albumin (Mn–curcumin/IRCOOH/albumin), and the size distribution is increased up to 84 nm according to TEM images (Fig. 1d).

In contrast, in an albumin-enriched environment (60 μM), Mn–curcumin/IRCOOH nanoparticles were reassembled into nanovesicles that could be entirely deconstructed, yielding a mixture of ultra-small nanoparticles and big nanovesicles (Fig. 1e). The visible shift in the size distribution of Mn–curcumin/IRCOOH nanoparticles confirmed its albumin response only exits in enough biomarkers.

We also assembled Mn–curcumin, IRCOOH NPs, curcumin/IRCOOH NPs, Mn–curcumin/IRCOOH NPs, and curcumin/IRCOOH/Mn(ii) NPs (adding Mn ions after co-assembling) to explain their smart albumin-response. These nanoparticles were prepared using the same methods as before, but the albumin was added right after the Mn–curcumin-based nanomaterials were formed. These assemblies uncover the mechanism that explains why Mn–curcumin/IRCOOH NPs are the only reaction to albumin over a critical concentration (Fig. 1f). The absorbance of Mn–curcumin (peaks at 432 nm and 498 nm) ranged from 293 nm to 635 nm. The IRCOOH NPs had obvious peaks at 707 nm and 852 nm. Curcumin/IRCOOH NPs and Mn–curcumin/IRCOOH NPs had similar peaks (420 nm, 778 nm, 851 nm), whereas curcumin/IRCOOH/Mn(ii) NPs were slightly red-shifted (435 nm, 485 nm, 779 nm, and 863 nm).

These results indicate that Mn(ii) ions was binding with curcumin, and the assembly mechanism of Mn–curcumin/IRCOOH NPs could be investigated with two building-block models like that of curcumin/IRCOOH, but increased stability.

**Fig. 2:**
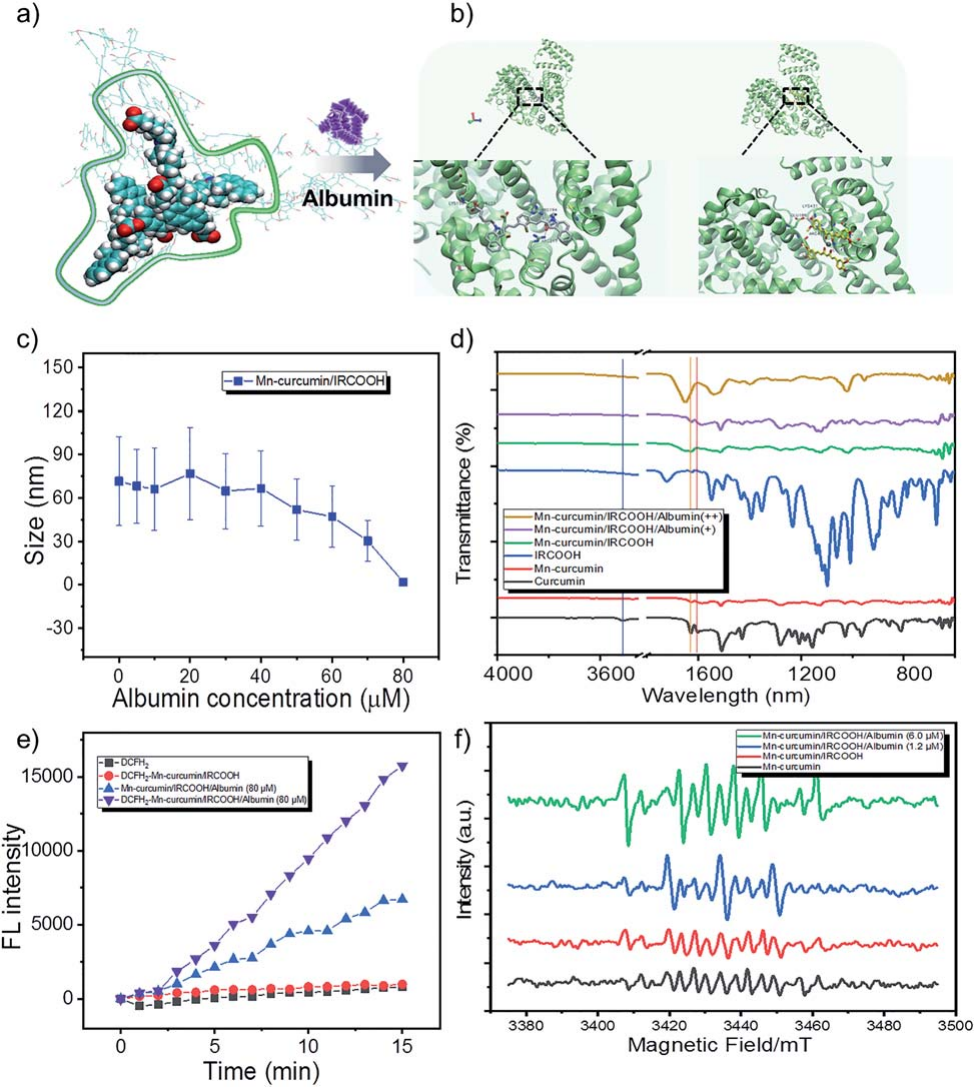
(a) and (b) Assembly patterns of activatable nanozymes varied from ROS scavenging to ROS generation. (c) The size distribution changed with increased albumin concentration from 0 to 80 μM. (d) FTIR and (e) albumin-mediated ROS generation of the activatable nanozyme. (f) Albumin-mediated radical generation of the ESR spectrum. Note: the purple line means –OH stretching (at 3516 cm^−1^); the orange line means C=O stretching vibrations at 1627 cm^−1^; and red line means C=O stretching vibrations at 1604 cm^−1^.

Thus, the inherent assemble model is reduced as Mn–curcumin and IRCOOH building blocks. In the whole of molecular dynamics (MD) simulation, it was performed using Gromacs (Version 5.1.4) package. The force field of Mn–curcumin complex and IRCOOH was generated by antechamber program in Ambertools18 package and acpype.py program, among which the bonded force field of metal-ligand was constructed by VFFDT program. The force field of protein was amber03. The atomic charges of the two molecules were fitted by DFT calculation under the restrained electrostatic potential (RESP) formalism and the resp program in Ambertools18. Water molecule was modelled using the tip3p potential. All solution models were firstly minimized utilizing the conjugate-gradient algorithm, and then equilibrated through running for 500 ps NVT simulations followed by 500 ps NPT simulations. Production runs in the NPT ensemble were then run for 150 ns at 298 K and 1 bar, employing the leapfrog algorithm with a time step of 2 fs to integrate the equations of motion. The electrostatic forces were treated with the particle-mesh Ewald approach. Both the cutoff values of van der Waals forces and electrostatic forces were set to be 1.2 nm. The LINCS algorithm was utilized to preserve bonds. According to molecular dynamics (MD) simulation and molecular docking computations, and the fluorescence spectrum of the co-assemblies (Fig. 2a, b, S9 and S10†), in the detailed investigation of the activatable Mn–curcumin/IRCOOH NPs, the Mn–curcumin/IRCOOH NPs possessed higher bond strengths of non-covalent interactions at high albumin concentration (90 μM) than other assembled nanomaterials. The changes in multiple synergetic intermolecular interactions of stacking models responsible for nanoparticle formation were confirmed (Fig. 2a). In Fig. S11 and S12,† the spectral information of IRCOOH with albumin is obviously different from the Mn–curcumin/IRCOOH with albumins. It means the other components will affect the fluorescence properties of IRCOOH, and it also proves the stability of Mn–curcumin/IRCOOH NPs too. Meanwhile, in Fig. 2c, the size distribution of Mn–curcumin/IRCOOH NPs showed an initial albumin-mediated step–step reduction. It proves Mn–curcumin/IRCOOH NPs are also albumin responsive and could dissemble into monomers too.

Due to binding interactions of Mn–curcumin and IRCOOH with bovine serum albumin,^67–69^ curcumin and cyanine both have a high affinity to albumin.^56,70,71^ The albumin model contains three domains for binding of various substrates (*e.g.* domain II of drug binding site I, domain III of drug binding site II). We have conducted the molecular docking computations, which were performed by Autodock 4.2.6 package. The albumin structure 4OR0 was employed from Protein Data Bank. Briefly, polar hydrogens and Kollman charges were added to the albumin. The ligands Mn–curcumin and IRCOOH were made torsion free by Autodock Tools. The entire 4OR0 molecule was enclosed in a grid box with 80 × 80 × 100 grid points and a grid spacing of 0.375 Å. Both 4OR0 and the ligands were kept rigid because the ligands had no flexible bonds topologically. The docking was performed using Lamarckian Genetic algorithm with 50 iterations and was repeated 10 times to generate 500 docking conformations of the ligands on the albumin. Results were clustered with a root-mean-square distance (RMSD) of 2.0 Å. Clusters with the lowest binding energies were considered the most favourable conformations. The final calculated result, Fig. 2b shows that NPs containing Mn–curcumin and IRCOOH were priory captured by albumin in domain II and in domain III, respectively. The phenol AOH group of Mn–curcumin was anchored by Glu 186, which is to induce ROS-scavenging.

In the Fourier transform infrared (FTIR) spectroscopy, the curcumin and Mn–curcumin/IRCOOH/albumin exhibits –OH stretching (at 3516 cm^−1^) and C=O stretching vibrations (at 1627 cm^−1^ and 1604 cm^−1^), which proves that the phenol AOH group is anchored at albumin and recovered its activity. It was further confirmed that the Mn–curcumin and cyanine (IRCOOH) as two monomers without aggregation-induced quenching were anchored in the different cavities sustained (Fig. 2b and d and S10, S12†).

In an *in vitro* study, we used dichlorodihydrofluorescein diacetate (DCFH_2_-DA) (10 μM) to directly detect ROS in aqueous solution. Due to disassemble of Mn–curcumin/IRCOOH in 80 μM albumin and the existence of unimolecular Mn–curcumin here (Fig. 2c and S11†), the spectral overlapping area between the Mn–curcumin and DCFH_2_-DA shows that the increase of fluorescence intensity in Mn–curcumin/IRCOOH-albumin (80 μM) group. But when we added the DCFH_2_-DA in this system, we could observe the fluorescence intensity of group DCFH_2_–Mn–curcumin/IRCOOH-albumin (80 μM) is 2 times higher than that of the control group (Mn–curcumin/IRCOOH-albumin (80 μM)) (Fig. 2e). Then, we identified albumin-mediated ROS scavenging^72,73^ and ROS generation of Mn–curcumin/IRCOOH NPs in the ESR spectrum too. The radical generation in Fig. 2f further supported activation of the phenol AOH group of nanozymes after initially scavenging ROS. As expected, the activated Mn–curcumin/IRCOOH NPs yielded the maximum amount of ROS through the assistance of albumin and light. It supports our hypothesis too. Thus, the system could serve as a dual bottom for further preclinical safety. Meanwhile, the oxidase-like mimic activated by the two switches under signal-on conditions showed potential for imaging-guided therapy. Based on this fact, we speculate that the produced monomer Mn–curcumin based on keto formation are more easily to transfer energy to oxygen than that in enol formation, and the fixation of phenol AOH group of Mn–curcumin in protein cavies helps generate more singlet oxygen for targeted phototherapy.

Interestingly, the activatable Mn–curcumin/IRCOOH NPs (50 μg mL^−1^) excited by a 655 nm laser (1.3 W cm^−2^, 10 minutes) also exhibited good photothermal properties. After three cycles of the photothermal test, it retains photothermal stability (Fig. S13†), suggesting its photostability as a good activatable antioxidant nanozyme in bioapplication.

**Fig. 3:**
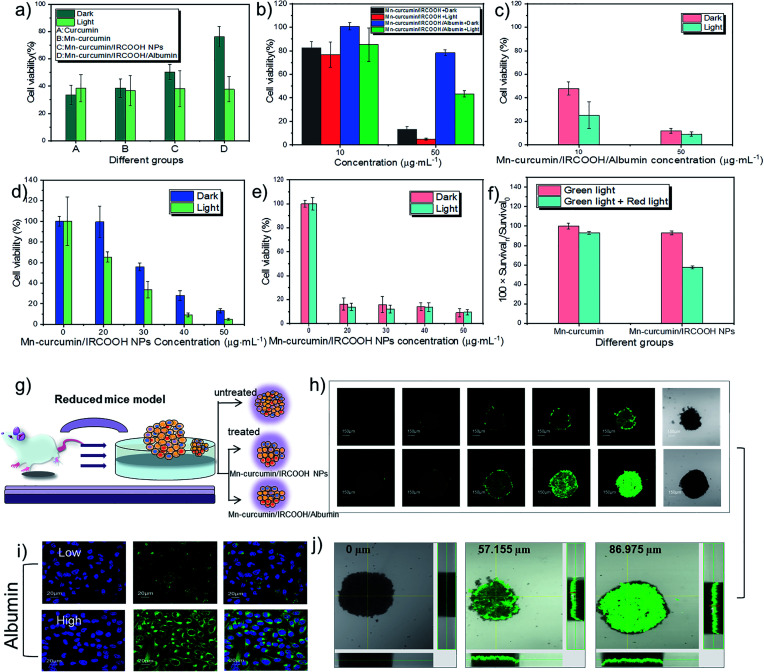
Albumin- and photo-mediated selectivity of nanomaterials in MTT assay in (a)–(f). (a) MTT assay of normal cells (L929 cells) treated with curcumin, Mn–curcumin, Mn–curcumin/IRCOOH NPs and Mn–curcumin/IRCOOH/albumin (4 hours, 50 μg mL^−1^). (b) The cell viability of L929 cells treated with Mn–curcumin/IRCOOH NPs and Mn–curcumin/IRCOOH/albumin (24 hours, 10 and 50 μg mL^−1^) and (c) the cell viability of HeLa cells treated with Mn–curcumin/IRCOOH/albumin (24 hours, 10 and 50 μg mL^−1^) (d) and (e) MTT assay of L929 cells and HeLa cells treated with varied Mn–curcumin/IRCOOH NPs concentrations (0–50 μg mL^−1^). (f) The effect of dual-light on HeLa cells. Survival_0_ represents the cells viability of HeLa cells treated with Mn–curcumin under green light. Survival_*n*_ represents the cell viability of HeLa cells treated with Mn–curcumin under single or dual light, and treated with Mn–curcumin/IRCOOH NPs under single or dual light. (g) In a reduced mouse model, (i) confocal images of albumin-mediated Mn–curcumin/IRCOOH NPs. (h) Imaging and (j) depth penetration of Mn–curcumin/IRCOOH NPs and Mn–curcumin/IRCOOH/albumin in a 3D tumour spheroid. Notice: (a) in the FBS-free medium, (b)–(f) in the FBS medium. The concentration without marks is recognized as Mn–curcumin concentration in nanomaterials.

The selectivity of the activated nanozyme was investigated through two cell models, the cancer cells model (HeLa cells) and the normal cells model (L929 cells). And the Fetal Bovine Serum (FBS) medium was utilized to mimic the concentration of albumin *in vivo*. In the absence of FBS in medium, which represents a low albumin concentration, curcumin, Mn–curcumin, Mn–curcumin/IRCOOH NPs, and Mn–curcumin/IRCOOH/albumin displayed noticeable differences in cell viability in the L929 cells. In the dark environment, when the Mn–curcumin/IRCOOH NPs surrounded by the albumin (Mn–curcumin/IRCOOH/albumin), it exhibits excellent biocompatibility than Mn–curcumin/IRCOOH NPs, Mn–curcumin, and curcumin. Their cell viability is 76.32%, 50.16%, 38.46% and 33.48%, respectively. After illumination, the cell viability of Mn–curcumin/IRCOOH/albumin is about 37.21%, which dramatically declines compared to the Mn–curcumin/IRCOOH NPs, Mn–curcumin, and curcumin (Fig. 3a).

When in the presence of FBS, normal cells (L929 cells) were treated with Mn–curcumin/IRCOOH NPs and Mn–curcumin/IRCOOH/albumin at the same concentration (50 μg mL^−1^ ), Fig. 3b exhibits that Mn–curcumin/IRCOOH/albumin reduced the cell cytotoxicity of Mn–curcumin/IRCOOH NPs. This evidence supports that the albumin in a suitable concentration could reduce the cell toxicity of Mn–curcumin/IRCOOH NPs, and it could help minimize the off-targeting side effects of normal cells too.

Because of the action of albumin on normal and cancer cells, all subsequent cell experiments are carried out in the presence of FBS. FBS contains albumin. So the Mn–curcumin/IRCOOH NPs in the FBS medium could be considered as partly Mn–curcumin/IRCOOH/albumin, whereas the Mn–curcumin/IRCOOH/albumin in the presence of FBS represents that they have much more albumin concentration than Mn–curcumin/IRCOOH NPs in this system.

At first, we tested the cell viability of HeLa cells treated with Mn–curcumin/IRCOOH/albumin (albumin concentration: 60 μM, Mn–curcumin/IRCOOH NPs: 10 μg mL^−1^, 50 μg mL^−1^) (Fig. 3b and c). After an increase in albumin concentration in the FBS medium, in comparison to the cell viability of L929 cells and HeLa cells, (dark: 78.29%, light: 43.16%), HeLa cells have an extremely high cancer cell inhibition (dark: 12.18%, light: 9.29%), suggesting that Mn–curcumin/IRCOOH/albumin shows the highest cell inhibition of cancer cells than the normal cells. We could say that the Mn–curcumin/IRCOOH NPs showed an obvious targeting ability to cancer cells over normal cells in the suitable albumin concentration. In the MTT assay, as the increase of Mn–curcumin/IRCOOH NPs concentration from 0 to 50 μg mL^−1^, after the treatment with 20 μg mL^−1^ of Mn–curcumin/IRCOOH NPs, the cell viability of L929 cells is decreased step by step while the cell viability of HeLa cells is decreased (dark: 99.53% *vs.* 16.39%; light: 65.16% *vs.* 13.91%). At the 50 μg mL^−1^ of Mn–curcumin/IRCOOH NPs, in comparison with L929 cells, the cell viability of HeLa cells treated with (50 μg mL^−1^) decrease obviously, which is even less than 10%. The selectivity of this activated nanozyme was further confirmed by the MTT assay (Fig. 3d and e).

The effect of single light and dual light on Mn–curcumin and Mn–curcumin/IRCOOH NPs was also investigated. In Fig. 3f, we used the cell viability of Mn–curcumin irradiated by single light as the control group. A 622 nm red laser (0.5 W cm^−2^, 2 min) was activated after a green LED (0.03 W cm^−2^, 30 min) aforementioned, and in comparison with the cell viability of HeLa cells treated by Mn–curcumin under dual light, the cell viability of HeLa cells treated by Mn–curcumin/IRCOOH decreased to 61.95%. This confirmed that the monomeric form of this activatable nanozyme also was responsive to the two wavelengths of light. In addition, before irradiated by red light, the cell viability of HeLa cells treated with Mn–curcumin/IRCOOH NPs (50 μg mL^−1^) and irradiated by green light illumination is about 9%. After irradiated by both green light and red light, the cell viability of HeLa cells treated with Mn–curcumin/IRCOOH NPs (50 μg mL^−1^) is super low (5%). It is accounted by the current cell viability of HeLa cells (9%) plus coefficient of light influence in cell viability (0.62). The super low cell viability (5%) of HeLa cells indicated their successful ablation. These results in Fig. 3 show the targeting ability of this photo-responsive nanozyme and its albumin-mediated singlet oxygen generation produces negligible side effects on normal tissues at special albumin concentrations, further suggesting its selectivity and decreased off-targeting capabilities. This activatable nanozyme has great potential for precise preclinical phototherapy with high therapy efficiency and selectivity. During the light-triggered treatment of HeLa cells but with ice packs in Fig. S14,† the cell viability of HeLa cells treated with Mn–curcumin/IRCOOH NPs (25 μg mL^−1^) + 8 μM albumin could show that the photothermal effect of Mn–curcumin/IRCOOH/albumin makes effects in the phototherapy. We speculate that the light-triggered photothermal effect and photodynamic effect are both important here.

In order to analyse the effects of oxygen concentration at a tumour site, we used a three-dimensional tumour model of a tumour spheroid as a reduced mouse model. It could mimic the behavioural characteristics of tumours and investigate prodrug-like nanozymes in detail (Fig. 3g). Comparing a “locked” nanozyme (Mn–curcumin/IRCOOH), an “unlocked” nanozyme (Mn–curcumin/IRCOOH/albumin, 60 μM) was easier uptake by the tumours during a short monitoring period (24 hours). Exposure to albumin showed activation of fluorescence of this nanozyme (100 μg mL^−1^) at the targeted site (Fig. 3i). At high albumin concentration, the complex achieves a high tumour penetration depth (57.155 μm *vs.* 86.975 μm), resided in the tumour for a long time, and was visualized well (Fig. 3h and j). These results indicate that the supramolecular prodrug-like nanozyme efficiently killed tumours cells, allowing preclinical imaging-guided phototherapy.

## Conclusions

In summary, we have developed adaptive nanozymes as a new strategy to overcome off-target toxicity with improved therapeutic efficiency. In the process, the nanozyme has an activatable dual enzyme-like activity. In the initial stage, it serves as a superoxide dismutase-like enzyme mimic that could provide the protective ability to consume excess ROS. A slight increase in albumin shows maintained stability, and in the albumin-enriched environment, the signal is activated at the tumour area as two monomers. Through this simple strategy, the nanozyme containing photosensitive drugs and photosensitizers achieves selective tumour cell ablation of nearly 100% under visualization, preventing off-targeting. Except for the reported biorthogonal bond-cleavage reaction of prodrug activation,^74^ such an adaptive nanozyme provides a synergetic activatable enzyme mimic strategy for early diagnosis and preclinical phototherapeutics.

## Data availability

Experimental details and additional data can be found in the ESI.†

## Author contributions

Jingjing Han: conceptualization, investigation, chemical synthesis, material characterization and preparation, *in vitro* test, software, writing – original draft, writing – review and editing. Haidong Li: chemical synthesis, writing – review and editing. Luyang Zhao: molecular simulation, writing – review and editing. Gyoungmi Kim: technical support. Yahui Chen: writing – review and editing. Xuehai Yan: conceptualization, funding acquisition, project administration, writing – review and editing, supervision. Juyoung Yoon: conceptualization, funding acquisition, project administration, writing – review and editing, supervision.

## Conflicts of interest

There are no conflicts to declare”.

## Supplementary Material

SC-013-D2SC02025D-s001
