# Supplementary material for: Albumin-mediated “Unlocking” of supramolecular prodrug-like nanozymes toward selective imaging-guided phototherapy
Source: Chem Sci. 2022 Jun 6;13(26):7814–20. doi: 10.1039/d2sc02025d (PMC9258398; doi:10.1039/d2sc02025d)
Supplement: SC-013-D2SC02025D-s001 [file SC-013-D2SC02025D-s001.pdf]

Electronic Supplementary Material (ESI) for Chemical Science.
This journal is © The Royal Society of Chemistry 2022

Albumin-mediated "Unlocking" of Supramolecular Prodrug-like Nanozymes Toward Selective Imaging-guided Phototherapy

Jingjing Han,[a] *Haidong Li,*[a,b] *Luyang Zhao,*[c] *Gyoungmi Kim,*[a] *Yahui Chen* [a,d]
*Xuehai Yan,** [c, e] *Juyoung Yoon**[a]

[a] Dr. J. Han, Dr. H. Li, Prof. J.Yoon. Department of Chemistry and Nanoscience, Ewha Womans University Seoul 03760 (Republic of Korea)

Email: jyoon@ewha.ac.kr, Website: http://of2m.ewha.ac.kr

[b] Dr. H. Li School of Bioengineering, Dalian University of Technology Dalian 116024, P.R. China.

[c] Dr. L.Zhao, Prof. X. Yan. State Key Laboratory of Biochemical Engineering Institute of Process Engineering, Chinese Academy of Sciences Beijing 100190, P.R. China.

Email: yanxh@ipe.ac.cn, Website: http: //www.yan-assembly.org

[d] Dr. Y.Chen, New and Renewable Energy Research Center, Ewha Womans University, Seoul 03760, Republic of Korea

[e] Prof. X. Yan. Center for Mesoscience, Institute of Process Engineering, Chinese Academy of Sciences, Beijing 100190, P.R. China.

Experimental Section

Materials

Curcumin, dichlorodihydrofluorescein diacetate ($DCFH_2$-DA), manganese(II) chloride, and (1,7-bis[4-hydroxy-3-methoxyphenyl]-1,6-heptadiene-3,5-dione) were purchased from Thermo (Invitrogen). N,N-Dimethylformamide (DMF) 6-Bromohexanoic acid, sodium acetate, acetic anhydride, $POCl_3$, cyclohexanone, DMF, and ethanol were purchased from Sigma-Aldrich. All chemicals were used as-is without further purification. Distilled de-ionized water (dd water) was prepared via laboratory ultrafiltration.

Characterization

Transmission electron microscopy (TEM) analysis was conducted on a JEM-2100F (JEOL) microscope at 100 kV. UV-visible absorption spectra were recorded with an Evolution 201 UV/vis spectrometer (Thermo Fisher Scientific) using a quartz cuvette. The particle size distribution in solution was measured via dynamic light scattering (DLS) using a Nanotrac Wave. Fluorescence spectra were recorded by a spectrofluorometer (Edinburgh FL900/FS900). Fourier transform infrared (FTIR) spectroscopy was performed using a VERTEX 80/80v FTIR spectrometer (BRUKER). The ^{1}H-NMR spectra were produced with a Bruker AM 300. Confocal laser scanning microscopy images were obtained using an Olympus Fluoview FV1200 confocal laser scanning microscope. A Bruker EMX EPR spectrometer equipped with a halogen lamp was used to investigate the ROS scavenging and ROS generation of samples. High-resolution mass spectrometry was performed on a Synapt G2-HDMS mass spectrometer (Waters, Manchester, U.K.), which was operated using MassLynx 4.1

software at KBSI (Korea Basic Science Institue, Ochang, Center of Research Equipment).

Synthesis of Mn-curcumin

The complex was synthesized by mixing curcumin with manganese (II) chloride at a molar ratio of 1:1 in methanol. Curcumin (0.74 g, 2 mmol) was dissolved in 50 mL pure methanol and heated at 60℃ under nitrogen. Manganese (II) chloride (0.406 g, 2 mmol) was added to the curcumin methanol solution, and the mixture was refluxed for 2 hours under nitrogen. The yellow solid product was filtered and washed with cold methanol and water to remove residual reactants; the product was dried under vacuum overnight.

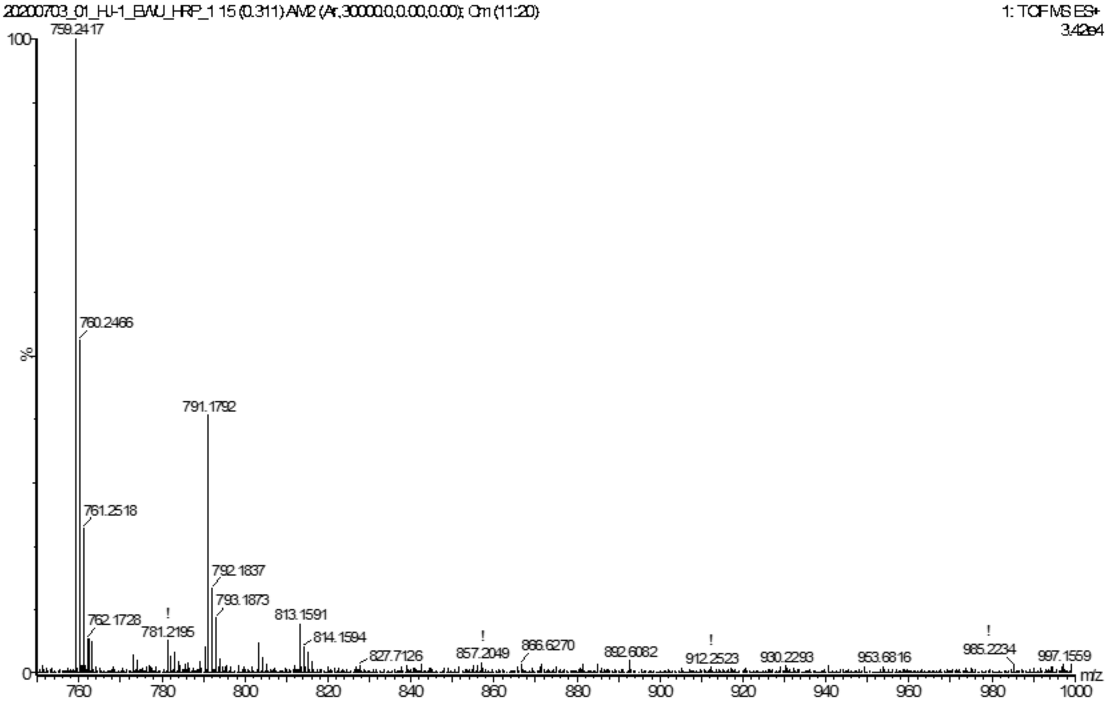

Figure S1. High resolution mass spectrum of Mn-curcumin.

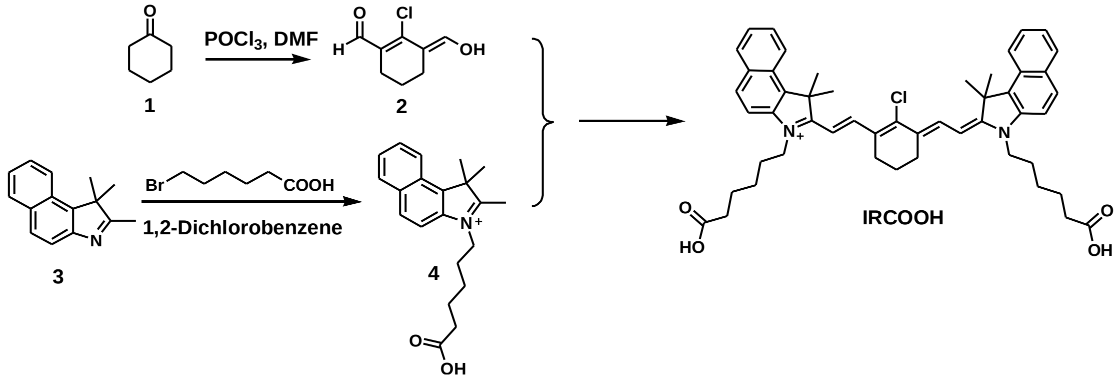

Figure S2. Synthesis of compound IRCOOH.

Synthesis of compound 4

Compound 2 was synthesized according to reference. (*Chem. Commun.*, 2014, ***50***, 1018-1020).

Synthesis and characterization of compound 4

Compound 3 (1.05g, 5 mM) and 6-bromohexanoic acid (970 mg, 5 mM) were added to 1,2-dichlorobenzene. The mixture was stirred at 130 ^{o}C for overnight and cooled down to room temperature. Then, compound 4 (1.21 g) was obtained by silica gel column chromatography. ^{1}H NMR (300 MHz, DMSO-d_6) δ: 8.37 (d, J = 8.4 Hz, 1H), 8.29 (d, J = 8.9 Hz, 1H), 8.25-8.20 (m, 1H), 8.15 (d, J = 8.9 Hz, 1H), 7.77 (dddd, J = 19.3, 8.0, 6.9, 1.3 Hz, 2H), 4.58 (t, J = 7.6 Hz, 2H), 2.94 (s, 3H), 2.24 (t, J = 7.1 Hz, 2H), 2.00-1.84 (m, 2H), 1.76 (s, 6H), 1.64-1.52 (m, 2H), 1.52-1.39 (m, 2H). ESI-HRMS: m/z calc'd. for $C_{21}H_{26}NO_2^+$ [M-Br]$^+$ 324.19581, found 324.19903.

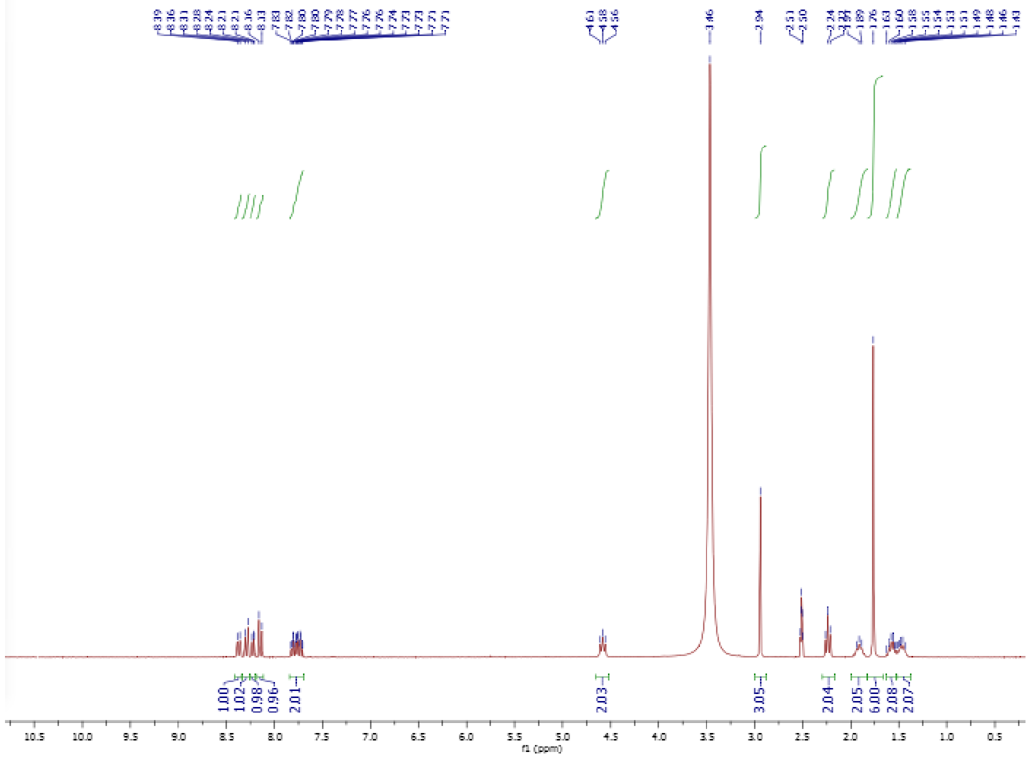

Figure S3. ^{1}H-NMR spectrum of compound 4 in DMSO-d_6.

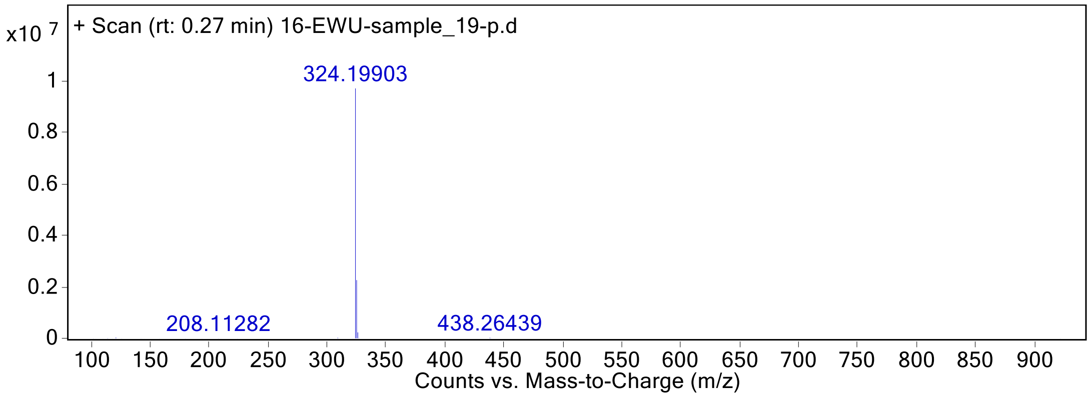

Figure S4. HRMS spectrum of compound 4.

Synthesis and characterization of IRCOOH

Compound 4 (806 mg, 2 mM), compound 2 (172 mg, 1 mM), sodium acetate (492 mg, 6 mM) were added to acetic anhydride (10 mL) under N_2 protection. The mixture was stirred at 130 ^{o}C for 1 hour and cooled down to room temperature. Finally, IRCOOH (542 mg) was obtained by silica gel column chromatography. ^{1}H NMR (300 MHz,

MeOD) δ: 8.54 (d, J = 14.2 Hz, 2H), 8.27 (d, J = 8.5 Hz, 2H), 8.01 (t, J = 9.3 Hz, 4H), 7.70-7.58 (m, 4H), 7.50 (t, J = 7.4 Hz, 2H), 6.33 (d, J = 14.2 Hz, 2H), 4.30 (t, J = 7.3 Hz, 4H), 2.77 (t, J = 5.7 Hz, 4H), 2.32 (t, J = 7.2 Hz, 4H), 2.02 (s, 12H), 1.99-1.84 (m, 6H), 1.80-1.64 (m, 4H), 1.64-1.45 (m, 4H). ESI-HRMS: m/z calc'd. for $C_{50}H_{56}ClN_2O_4^+$ [M-Br]$^+$ 783.3923, found 783.3906.

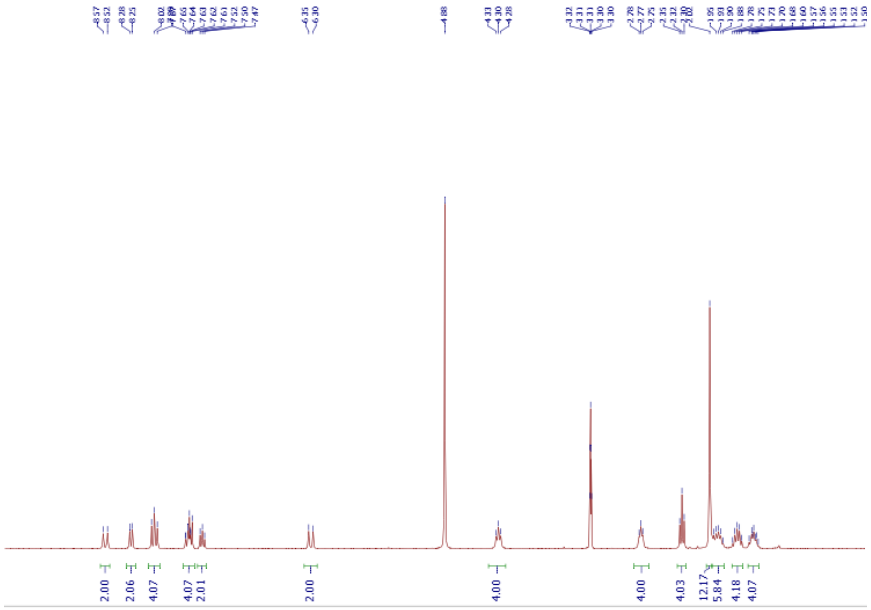

Figure S5. ^{1}H-NMR spectrum of compound IRCOOH in MeOD.

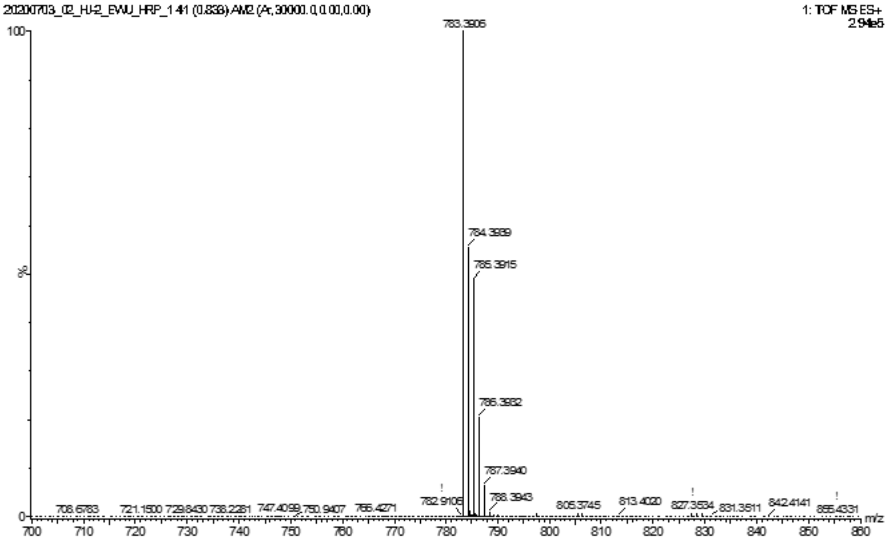

Figure S6. High resolution mass spectrum of the compound IRCOOH.

Preparation of Mn-Curcumin-based nanomaterials

First, IRCOOH (10 mg·mL^{-1}) and Mn-Curcumin (5 mg·mL^{-1}) were dissolved in a solution of dimethyl sulfoxide and ethanol, respectively. In a typical process, 10 μL IRCOOH (final concentration: 100 μg·mL^{-1}) was mixed with 50 μL Mn-Curcumin (final concentration: 250 μg·mL^{-1}) in 1 mL aqueous solution at a feed ratio of 1:5. Samples with other components (curcumin, IRCOOH, Curcumin/IRCOOH NPs, Mn-curcumin/IRCOOH NPs, and Curcumin/IRCOOH/Mn(II) NPs) were prepared using similar methods. Albumin was quickly added after the formation of Mn-curcumin-based nanomaterials.

Molecular dynamics simulation

The molecular dynamics (MD) simulation was performed using Gromacs (Version 5.1.4) package.[1] The force field of Mn-curcumin complex and IRCOOH was generated by antechamber program in Ambertools18 package[2] and acpype.py program[3], among which the bonded force field of metal–ligand was constructed by VFFDT program[4]. The force field of protein was amber03. The atomic charges of the two molecules were fitted by DFT calculation under the restrained electrostatic potential (RESP) formalism and the resp program in Ambertools18. Water molecule was modeled using the tip3p potential. All solution models were firstly minimized utilizing the conjugate-gradient algorithm, and then equilibrated through running for 500 ps NVT simulations followed by 500ps NPT simulations. Production runs in the NPT ensemble were then run for 150 ns at 298 K and 1 bar, employing the leapfrog algorithm with a time step of 2 fs to integrate the equations of motion. The electrostatic forces were treated with the particle-mesh Ewald approach. Both the

cutoff values of van der Waals forces and electrostatic forces were set to be 1.2 nm. The LINCS algorithm was utilized to preserve bonds.

Molecular docking computations

The molecular docking computations were performed by *Autodock 4.2.6* package. [5] The albumin structure 4OR0 was employed from Protein Data Bank.[6] Briefly, polar hydrogens and Kollman charges were added to the albumin. The ligands Mn-Curcumin and IRCOOH were made torsion free by *Autodock Tools*. The entire 4OR0 molecule was enclosed in a grid box with 80 × 80 × 100 grid points and a grid spacing of 0.375 Å. Both 4OR0 and the ligands were kept rigid because the ligands had no flexible bonds topologically. The docking was performed using Lamarckian Genetic algorithm with 50 iterations and was repeated 10 times to generate 500 docking conformations of the ligands on the albumin. Results were clustered with a root-mean-square distance (RMSD) of 2.0 Å. Clusters with the lowest binding energies were considered the most favorable conformations.

ROS generation after ROS loss

$DCFH_2$-DA (10 μM) was dissolved with DMSO and Mn-curcumin/IRCOOH (50 ug mL^{-1} Mn-curcumin, 20 ug mL^{-1}, IRCOOH) in the presence or absence of BSA (80 μM) and added to 1 mL of dd H_2O. Mn-curcumin/IRCOOH in the presence of BSA was the control group without $DCFH_2$-DA. For removal of oxygen, a mixed solution was bubbled with argon for 30 min. The reaction system was irradiated with a halogen lamp (100 mW cm^{-2}), and the decrease in adsorption at 526 nm was recorded via FL spectrophotometry.

Photothermal stability

An aqueous solution (1.0 mL) of Mn-curcumin/IRCOOH NPs (50 ug $\cdot$mL^{-1} Mn-curcumin, 20 ug $\cdot$mL^{-1}, IRCOOH) was irradiated with a 655 nm laser (1.3 W $\cdot$cm^{-2}, 10 minutes) to record its temperature.

Selectivity of nanomaterials in cancer cells and normal cells

HeLa cells (1×10^4 cells $\cdot$well^{-1}) and L929 cells (1×10^4 cells $\cdot$well^{-1}) were seeded in 96-well plates and allowed to attach for 24 hours. The L929 cells were treated with curcumin, Mn-curcumin, Mn-curcumin/IR-COOH nanoparticles, and Mn-curcumin/IRCOOH/albumin in FBS-free medium for 4 hours, while the L929 cells and HeLa cells were treated with Mn-curcumin/IR-COOH nanoparticles (0~50 μg $\cdot$mL^{-1}, 24 hours) in normal medium. They were washed with fresh culture medium and irradiated with a green LED at 0.03 W $\cdot$cm^{-2} for 30 minutes. After irradiation, the cells were incubated in the dark for 24 hours. As a control, we used the same conditions but without irradiation to compare cell viability. Apart from that, the L929 cells incubated with Mn-curcumin/IR-COOH nanoparticles or Mn-curcumin/IR-COOH/albumin (10 and 50 μg $\cdot$mL^{-1}) were cultured in a dark and light environment (24 hours), whereas HeLa cells were treated only with Mn-curcumin/IR-COOH/albumin. After illumination (a green LED, 0.03 W $\cdot$cm^{-2} $\cdot$30 min), the HeLa cells treated with Mn-curcumin/IR-COOH nanoparticles (50 μg $\cdot$mL^{-1}) were illuminated with an additional red LED light (0.5 W $\cdot$cm^{-2} $\cdot$2 min). Following these treatments, standard MTT assays were conducted. For this, a 10% MTT (5 mg $\cdot$mL^{-1}) solution was added to each well for 4 hours. Finally, 150 μL of DMSO was pipetted into the medium, followed by absorption measurements (490 nm) on a microplate reader (Thermo Fisher Scientific). Cell viability was defined as the

percentage of live cells per total number of cells. Note: the concentration here is based on the Mn-curcumin in Mn-curcumin/IRCOOH nanomaterials.

Determination of photodynamic and photothermal effect in cancer cell death

HeLa cells (1×10^4 cells $\cdot$well^{-1}) were seeded in 96-well plates and allowed to attach for 24 hours. To minimize the cancer cell's inhibition and better to observe the photodynamic and photothermal effect in cancer cells, the HeLa cells treated with Mn-curcumin/IR-COOH/Albumin (50 μg $\cdot$mL^{-1} for Mn-curcumin, 20 μg $\cdot$mL^{-1} for IRCOOH, 8 μM for Albumin) are only for 12 hours in a normal medium. Then, HeLa cells are separated into four groups (A, B, C, and D). Group A with no treatment, Group B is incubated with Mn-curcumin/IR-COOH/Albumin, Group C and Group D are incubated with Mn-curcumin/IR-COOH/Albumin and are irradiated by a green LED (0.03 W cm^{-2} $\cdot$30 min) too, whereas group C use an ice pack to decrease heat. Following these treatments, we allow the HeLa cells to grow up for another 24 hours. Finally, standard MTT assays were conducted.

Reduced mouse model

We coated inner plate wells with 60 μL of 1.5% agarose. After cooling the agarose solution to room temperature in 20 minutes, 3D multicellular tumor spheroids were prepared with HeLa cells in the aforementioned ultra-low attachment 96-well plate (Corning) at a density of 5000 cells per well in 100 μL of culture medium. 3D cell spheroids were incubated at 37 ℃ under a 5% CO_2 atmosphere. After the spheroids formed over 3 days, they were processed and incubated with Mn-curcumin/IRCOOH or Mn-curcumin/IRCOOH/Albumin for 24 hours at the desired concentration (50 μg·mL^{-1}) and washed twice with PBS. They were subsequently transferred to a

confocal dish and imaged at different depths (*z*-stacking) with a laser scanning confocal fluorescence microscope.

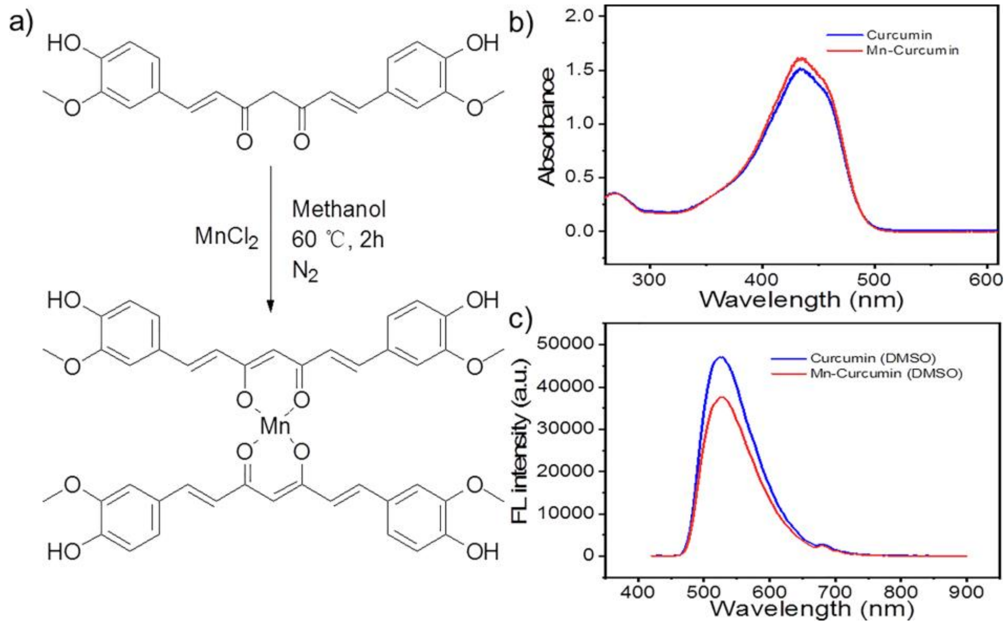

Figure S7. a) Synthesis of the complex Mn-curcumin. b) and c) UV spectra and FL spectra of curcumin and Mn-curcumin- complex in DMSO at the concentration of 25 ug·mL^{-1}. In the Visible-UV spectrum, the absorbance of Mn-curcumin has a little increase and blue-shift at peak 433 nm and 456 nm, while the absorbance of curcumin is still at 435 nm and 457 nm. Meanwhile, after binding with Mn(II) ion, the charge transfer from the curcumin to Mn (II) ions makes it behave a decrease of FL intensity in the spectrum, as well a red-shift from 524 nm to 530 nm. These results prove that we successfully got the Mn-curcumin complex.

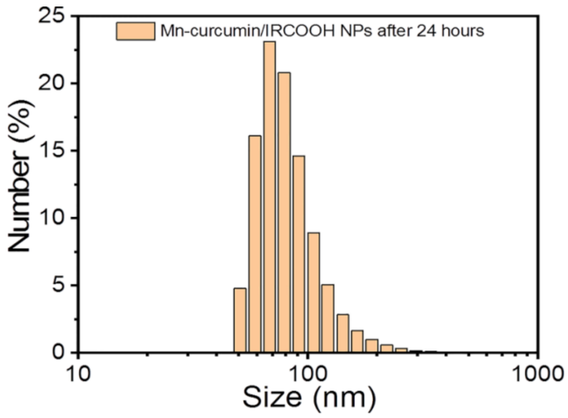

Figure S8. The size distribution of Mn-curcumin/IRCOOH NPs after 24 hours, measured by DLS.

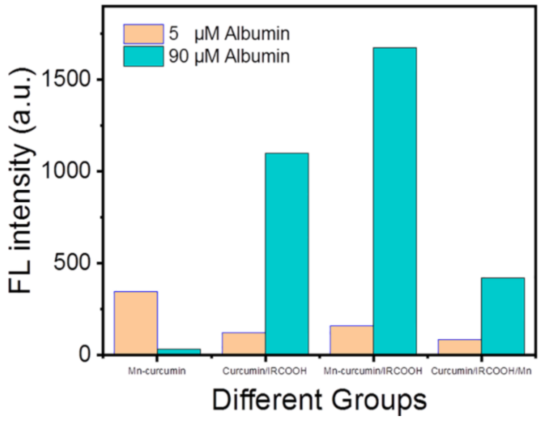

Figure S9. The Fluorescence intensity of Mn-curcumin, Curcumin/IRCOOH NPs, Mn-curcumin/IRCOOH NPs, and Curcumin/IRCOOH/Mn (II) NPs with 5 μM and 90 μM albumin.

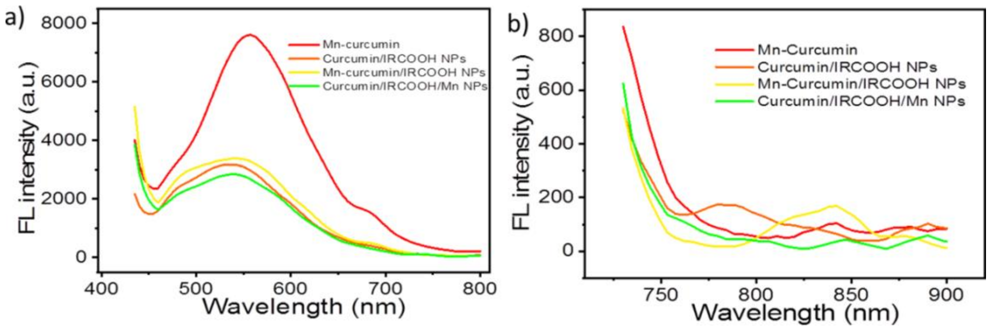

Figure S10. a) and b) Fluorescence intensity of Mn-curcumin, IRCOOH NPs, Curcumin/IRCOOH NPs, Mn-curcumin/IRCOOH NPs, and Curcumin/IRCOOH/Mn(II) NPs (adding Mn(II) ions after co-assembling) in aqueous solution at the concentration of 25 ug ·mL^{-1} curcumin and Mn-curcumin, 10 ug ·mL^{-1} IRCOOH.

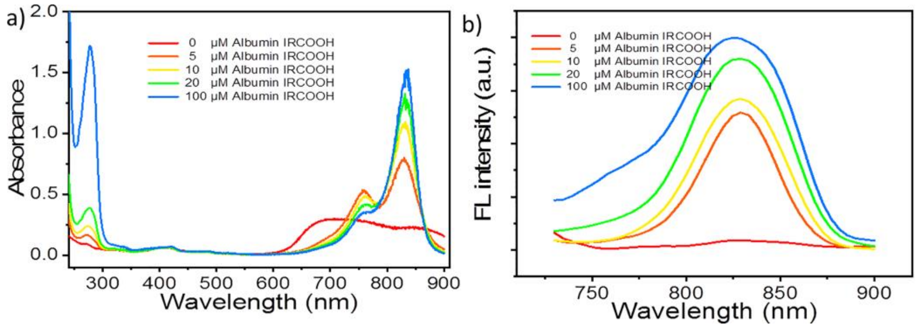

Figure S11. a) UV spectra and b) FL spectra of IRCOOH nanoparticles (10 ug ·mL^{-1}) in the system with various Albumin (0, 5, 10, 20, 100 μM), and the IRCOOH with 0 μM Albumin is regarded as one control group.

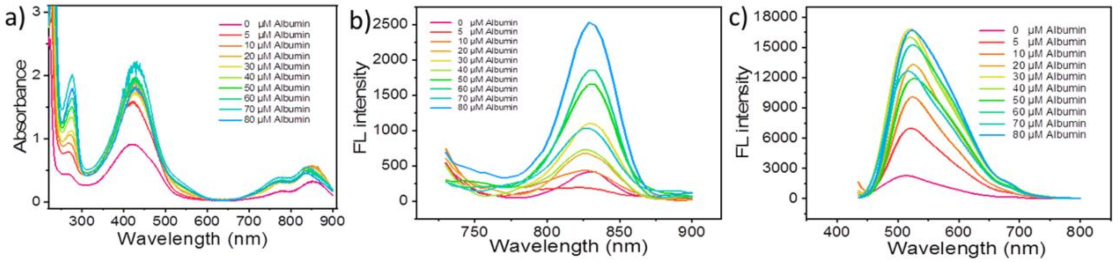

Figure S12. a) UV spectra and b), c) FL spectra of Mn-curcumin/IRCOOH NPs (50 ug ·mL^{-1} Mn-curcumin, 20 ug ·mL^{-1}, IRCOOH) in the different albumin concentration of albumin, and the Mn-curcumin/IRCOOH NPs with 0 μM Albumin is regarded as control groups.

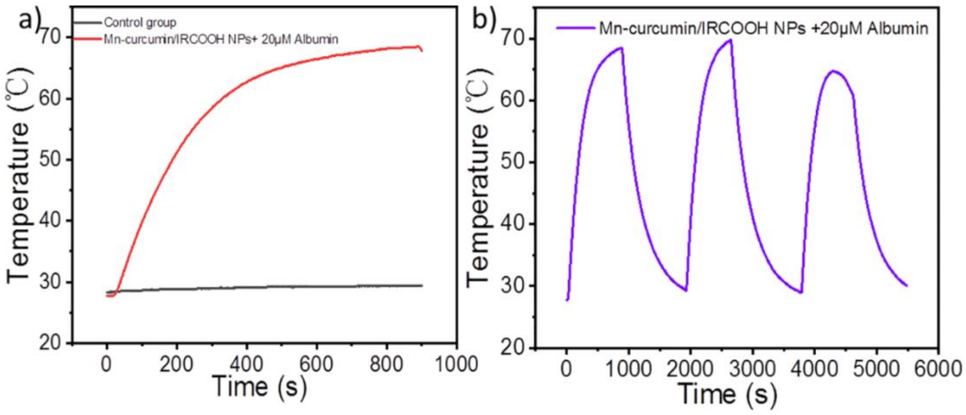

Figure S13. a) and b) The PTT effect of Mn-curcumin/IRCOOH NPs (50 ug ·mL^{-1} Mn-curcumin, 20 ug ·mL^{-1}, IRCOOH). Compared with the control groups (water), the Mn-curcumin/IRCOOH NPs with albumin (20 μM) can have good photothermal stability.

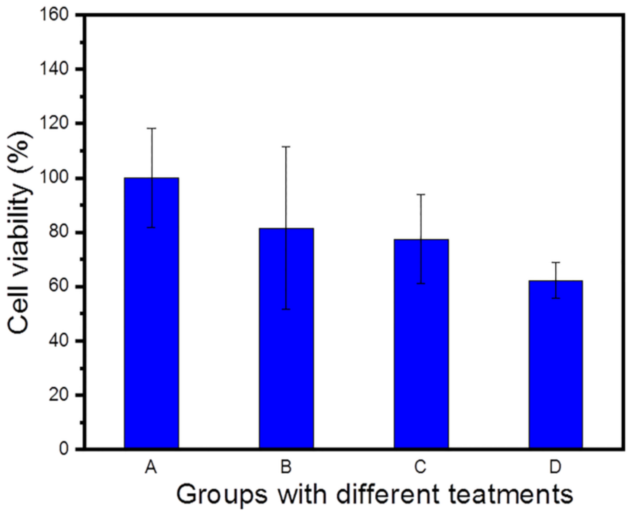

Figure S14. The cell viability of HeLa cells with different treatments. Group A is control group with no treatment, group B is incubated with Mn-curcumin/IRCOOH (25 ug ·mL^{-1} Mn-curcumin, 10 ug ·mL^{-1}, IRCOOH) + 8 μM Albumin for 12 hours. The groups C and D are both incubated with same samples and irradiated by green LED (0.03 W cm^{-2}, 30 minutes), whereas the group C decreases its' heat under illumination.

References for SI

[1] M. J. Abraham, T. Murtola, R. Schulz, S. Pall, J. C. Smith, B. Hess, E. Lindahl, *SoftwareX*, 2015, 1, 19–25.

[2] D.A. Case, I.Y. Ben-Shalom, S.R. Brozell, D.S. Cerutti, T.E. Cheatham, III, V.W.D. Cruzeiro, T.A. Darden, R.E. Duke, D. Ghoreishi, M.K. Gilson, H. Gohlke, A.W. Goetz, D. Greene, R Harris, N. Homeyer, Y. Huang, S. Izadi, A. Kovalenko, T. Kurtzman, T.S. Lee, S. LeGrand, P. Li, C. Lin, J. Liu, T. Luchko, R. Luo, D.J. Mermelstein, K.M. Merz, Y. Miao, G. Monard, C. Nguyen, H. Nguyen, I. Omelyan, A. Onufriev, F. Pan, R. Qi, D.R. Roe, A. Roitberg, C. Sagui, S. Schott-Verdugo, J. Shen, C.L. Simmerling, J. Smith, R. SalomonFerrer, J. Swails, R.C. Walker, J. Wang, H. Wei, R.M. Wolf, X. Wu, L. Xiao, D.M. York, P.A. Kollman, 2018, AMBER 2018, University of California, San Francisco.

[3] J. Wang, W. Wang, P. A. Kollman, D. A. Case, *J. Mol. Graph. Model*. 2006, 25, 247–260.

[4] S. Zheng, Q. Tang, J. He, S. Du, S. Xu, C. Wang, Y. Xu, Fu Lin, *J. Chem. Inf. Model.*2016, 56, 4, 811–818.

[5] G. M. Morris, R. Huey, W. Lindstrom, M. F. Sanner, R. K. Belew, D. S. Goodsell, A. J. Olson, *J. Comput. Chem.* 2010, 12, 2785–2791.

[6] H. M. Berman, J. Westbrook, Z. Feng, G. Gilliland, T. N. Bhat, H. Weissig, I. N. Shindyalov, P. E. Bourne, The Protein Data Bank. *Nucleic Acids Res.* 2000, 28, 235–242.
